# Risk Assessment of AFM1 in Raw Milk and Dairy Products Produced in Armenia, a Caucasus Region Country: A Pilot Study

**DOI:** 10.3390/foods13101518

**Published:** 2024-05-13

**Authors:** Davit Pipoyan, Astghik Hovhannisyan, Meline Beglaryan, Alberto Mantovani

**Affiliations:** 1 Center for Ecological-Noosphere Studies of NAS RA, Abovyan 68, Yerevan 0025, Armenia; david.pipoyan@cens.am (D.P.); astghik.hovhannisyan@cens.am (A.H.); 2 Italian National Food Safety Committee, Lungotevere Ripa 1, 00153 Rome, Italy; alberto.mantovani1956@gmail.com

**Keywords:** mycotoxins, aflatoxin M1, daily intake, exposure assessment, risk

## Abstract

This paper presents the first assessment of dietary exposure to aflatoxin M1 (AFM1) and associated health risks through milk and dairy product consumption in Armenia. Data on AFM1 in raw milk were obtained from an annual residue monitoring program. Additionally, commonly consumed dairy products (pasteurized milk, cheese, sour cream, curd cheese) were sampled, considering the sources of raw milk used by dairy companies. Per capita consumption of raw milk was sourced from national food balance databases, while individual consumption data for dairy products was collected via a 24 h recall survey with 1400 adult respondents. Detectable levels of AFM1 were observed in 7.14% of raw milk samples (up to 0.334 μg/kg) and, albeit at lower amounts (up to 0.009 µg/kg), in 30% and 40% of sour cream and curd cheese, respectively. The AFM1 levels were lower than the national maximum permitted level (0.5 μg/kg); however, levels in raw milk exceeded the EU ML (0.05 μg/kg). The estimated margin of exposure values for dairy products indicated no significant risk, whereas a reasonable worst-case estimate, using the measurable levels of AFM1 in raw milk consumption indicated a potential public health concern. This study provides a scientific basis for evaluating aflatoxin issues in the Caucasus area.

## 1. Introduction

Milk and dairy products can play notably important roles in human nutrition as concentrated sources of macro- and micronutrients [1,2,3]. In many countries across Europe, the Middle East, and Central Asia, milk and dairy products are substantial sources of proteins, fats, minerals, and vitamins due to their high and widespread consumption. Indeed, in the coming decade, the global per-capita consumption of dairy products is expected to increase by 1.0% per annum [4]. Thus, assessing and managing the safety issues of dairy foods is important in order to fully exploit their benefits for nutritional security. 

Mycotoxins are toxic secondary metabolites of different funguses, mainly of the genera Aspergillus, Fusarium, and Penicillium, which commonly infect either plants used for human and/or animal food and improperly stored feeds and foods: mycotoxins are known to cause serious, usually chronic, adverse health effects to humans and animals consuming the affected commodities [5,6]. Aflatoxins are some of the most toxic mycotoxins, with Aflatoxin B1 (AFB1) being the most important. In particular, aflatoxins contaminate corn, other cereals, and nuts; these toxins are hepatotoxic and can induce liver cancer [7,8,9,10,11,12]. They are produced by certain types of fungi (e.g., *Aspergillus flavus* and *Aspergillus parasiticus*) that grow on soil, decaying vegetation, hay, and grains [13]. According to an assessment published by the EFSA in 2020, due to widespread exposure and high toxicity, AFB1 and other aflatoxins are health concerns for consumers in the European Union [7]. In particular, the EFSA established a benchmark dose (BMLD) of 0.4 μg/kg body weight/day for AFB1; for chronic risk assessment, the EFSA established a standard margin of exposure (MOE) of 10,000 for genotoxic carcinogens, such as aflatoxins. According to the EFSA’s estimates, the MOEs have been consistently below 10,000 across different European Countries and age groups, hence highlighting a health concern [7]. Dairy products, being foods of animal origin, are especially susceptible to aflatoxin exposure; the contamination of animal feeds can lead to the presence of aflatoxin M1 (AFM1) [14,15], which is the hydroxylated metabolite of AFB1 excreted in milk by cattle and other ruminants [7,16,17]. The presence of aflatoxin M1 is influenced by factors related to climate, feed manufacturing, and animal husbandry [18,19]. According to EFSA, AFM1 is a liver carcinogen like the parent compound B1, albeit less potent; the health-based guidance value for chronic risk assessment is 4 μ/kg bw per day [8]. Like other aflatoxins, AFM1 has strong thermal stability, even at a high temperature, which creates an obstacle regarding its reduction in milk and dairy products; in fact, research shows that pasteurization, domestic cooking, and other thermal treatments are poorly effective [6,13,20]. Aflatoxin M1 is bound to milk proteins; hence, it can concentrate in fresh (especially ripened) cheeses [21]. AFM1 is the only aflatoxin metabolite for which the maximum residue levels were set separately. Regulatory bodies, including the EU, Codex Alimentarius, and the US Food and Drug Administration, have established maximum residue levels for AFM1 in milk and dairy products. In EU countries, for milk and dairy products, the maximum level is set at 0.05 μg/kg [22,23], while 0.5 μg/kg is the level set by Codex Alimentarius [24] and the US Food and Drug Administration standards [25]. Among countries in the Caucasus area, in Iran, the national standard is set at 0.1 μg/L [26].

Dairy production plays a crucial role in ensuring food security and promoting societal well-being across the entire Caucasus area [27], particularly in Armenia, where dairy farming is one of the well-developed agricultural sectors. According to the Statistical Committee of the Republic of Armenia, in 2021, milk production was about 670,700 tons, providing a national self-sufficiency of 87.7%. Meanwhile, the consumption of milk per capita slightly decreased (247.1 kg/year) compared to 2019 (256.8 kg/year) and 2020 (258.1 kg/year) [28]. The safety issues in dairy production are regulated by the Technical Regulations of the Customs Union [29,30] and the maximum allowable level of AFM1 for dairy products is set at 0.5 μg/kg. Despite the importance of dairy production, the mycotoxin contamination issue in Armenian dairy products has not been thoroughly investigated and the real situation involving mycotoxins in dairy products is not known. Therefore, this study provides a pilot assessment of AFM1 exposure and associated health risks through the consumption of selected dairy products by Armenia’s adult population. This pilot study mainly highlights whether a problem exists; therefore, we mainly focus on raw milk as the primary marker of AFM1 exposure in feeds and farms, as well as a source of contamination for dairy products. Meanwhile, considering the peculiarities of dairy production in the country, particularly the fact that Armenian dairy-producing companies collect and utilize raw milk from various regions of the country, this study also focuses on commonly consumed dairy products such as pasteurized milk, cheese, sour cream, and curd cheese. To conduct this pilot risk assessment study, data from an annual residue monitoring program on raw milk were used as the most efficient tools to identify potential health concerns. Given that Armenia applies an individual-based approach to dietary investigations and considering the similarities in consumption patterns and milk supply chains across the Caucasus region, this study’s outcomes are expected to provide important insights into neighboring countries as well.

## 2. Materials and Methods

### 2.1. Raw Milk Sampling and Analysis

Raw cow milk sampling was conducted in 2021 within the framework of the national residue monitoring program on residues in animal-origin food products by the Food Safety Inspection Body (FSIB) of Armenia. The samples were collected from dairy farms randomly selected across 10 regions (also known as “marzes” in Armenia): Kotayk, Armavir, Lori, Shirak, Aragatsotn, Ararat, Vayots Dzor, Syunik, Tavush, Gegharkunik. In total, 42 samples of raw milk (each weighing approximately 500 mL or g) were collected and analyzed for AFM1.

After collection, samples were transported to the Republican Veterinary-Sanitary and Phytopathology Center of Laboratory Service (RVSPCLS) SNCO and stored at 4–8 °C until analyzed.

Detection of AFM1 residues in milk samples was carried out using ELISA as a routine screening based on the MaxSignal^®^ Aflatoxin M1 ELISA Kit (PerkinElmer, Austin, TX, USA). The sample preparation was conducted following the manufacturer’s manual guidelines from the ELISA kit [31]. The washing station, Thermo Scientific™ Wellwash™ (Thermo Fisher Scientific Inc., Vantaa, Finland), was used for strip washing. The ELISA rider Multiskan™ FC (Thermo Fisher Scientific Inc., Vantaa, Finland) was used to observe the residue absorbents at an optical density of 450 nm (OD450). The absorbance results were treated with spatial programs provided by manufacturers: MaxSignal^®^ ELISA analyses program in Microsoft Excel 2016 and RIDASOFT^®^ Win.NET (version 1.98) were used.

The screening for AFM1 was followed by further confirmation and quantification through a standardized diagnostic method. Therefore, the ELISA results were confirmed with liquid chromatography with tandem mass spectrometry (LC-MS/MS) [32] for a more accurate quantification of AFM1 residues. The LC-MS/MS system was equipped with a fluorescence detector. For LC-MS/MS, all the reagents and solvents used were of HPLC grade, such as acetonitrile and methanol Carlo Erba (Carlo Erba reagents S.A.S., Val de Rueil, France). The limit of quantification (LOQ) was 0.023 µg/kg for AFM1.

### 2.2. Dairy Product Sampling and Analysis

The sampling of locally produced and commonly consumed products (pasteurized milk, cheese, sour cream, and curd cheese) was conducted randomly and involved the main dairy producer companies, which hold a relatively considerable market share in the country’s dairy sector. Therefore, 10 main producers have been considered, and a total of 40 samples of pasteurized milk, cheese, sour cream, and curd cheese (10 samples of each dairy product) were collected from several supermarkets.

The samples of dairy products were analyzed for AFM1 in a “Standard Dialog” LLC Laboratory using HPLC (Shimadzu LC-2010C, Kyoto, Japan) and following the methodology of the interstate standard “GOST 30711-2001: Food products: Methods for the detection and determination of the content of B and M aflatoxins” [33]. All the reagents and solvents used were of HPLC grade. The limit of quantification (LOQ) was 0.001 µg/kg for AFM1.

### 2.3. Consumption Data Collection and Statistical Analysis

The data on the per capita consumption of raw milk were obtained from a database on national food balances [28], while individual-based consumption data for dairy products were collected through a 24 h recall survey [34]. The survey was conducted in 2021 among Armenia’s adult population (18–80 years old) as part of the State program 20TTCG-4A001. Data were collected from all 11 marzes (regions) in Armenia, with 1400 adult respondents (females n = 734, males = 666) participating.

The variance and normality of survey data were identified by the ANOVA test and SPSS software (IBM SPSS, v.22) was used. The final calculations were conducted with the help of MS Office Excel software (Microsoft Excel 2016).

### 2.4. Daily Intake and Risk Assessment of AFM1

Daily intake (DI) of AFM1 was calculated by Equation (1) [35], as follows:

DI = C × IR/BW(1)

where C is the aflatoxin content in the sample (µg/kg); IR is the ingestion rate, i.e., the average daily consumption of the product by the Armenian adult population (kg/day); and BW is the average body weight of adult females and males in Armenia. Based on the 24 h recall survey data analysis, the average weight for Armenia’s adult population was 71.5 kg, while for females and males, the average weight values were 66.1 kg and 77.5 kg, respectively. These data were used in further calculations.

The International Agency for Research on Cancer (IARC) declared aflatoxins, including AFM1, as genotoxic carcinogens [36]; hence, to calculate health risks, the MOE approach was used with Equation (2) [35], as follows:

MOE = HBGV/DI(2)

where HBGV denotes the up-to-date health-based guidance values of AFM1, the BMLD_10_ of 4 µg/kg per day.

For the above-mentioned calculations, the average of the detected AFM1 content was used. For the studied samples with non-detected (ND) contents of AFM1, the standard approach for handling left-censored data (i.e., <LOD or LOQ) in the dietary exposure assessment [37,38,39] was applied. Therefore, in the lower bound (LB), the results below the LOQ were replaced with zero, and in the upper bound (UB), the results below the LOQ were replaced with a value equal to LOQ. Additionally, as a point estimate between the two extremes, the middle bound (MB) scenario was considered by assigning a value of LOQ/2 to the left-censored data (i.e., <LOQ) reported in this study.

## 3. Results and Discussion

### 3.1. The Contents of AFM1 in Raw Milk

AFM1 was detected in 7.14% (3 out of 42) of the raw milk samples (Table 1). It is important to note that even though the detected contents of AFM1 in raw milk were below the allowable level (0.5 µg/kg) set by the Technical Regulation applicable in Armenia [29], they exceeded the maximum level of 0.05 µg/kg set by the Commission Regulation (EU) 2023/915 [23] by 1.8, 3, and 6.7 times.

The concentrations of AFM1 in milk have been reported from different countries in the Mediterranean part of the EU and the Middle East, which share some similarities with Armenia concerning environmental and dietary patterns (Table 2).

A comparison with the data from neighboring countries, Iran and Turkey, as well as data from Lebanon (Table 2), indicate that the mean concentrations of AFM1 in raw cow milk were consistently lower, while the rate of positive samples was higher than in our study (Table 1). Additionally, it is worth mentioning that in this study, high-level AFM1 residues were detected only in a minority of samples (3 out of 42, 7.1%) of raw milk samples. A possible explanation is the occurrence of “spots” of feed contamination with aflatoxins; in fact, feed is a major source of the presence of AFM1 in milk [7,14].

### 3.2. The Contents of AFM1 in Dairy Products

Among the studied dairy products, AFM1 was not detected in the samples of pasteurized milk and cheese (Table 3). However, in the case of sour cream (n = 10) and curd cheese samples (n = 10), 30% and 40%, respectively, had detectable contents of AFM1, which were below the allowable level (0.5 µg/kg) set by the Technical Regulation applicable in Armenia [29], and the maximum level (0.05 µg/kg) set by the Commission Regulation (EU) 2023/915 [23].

In contrast to pasteurized milk produced in Armenia, milk products, including heat-treated milk from Mediterranean countries (Greece, Spain, and Lebanon) [41,42,43], contained detected levels of AFM1, as shown in Table 4. These reported levels of AFM1 were higher than those detected in dairy products from Armenia (Table 3).

It is worth mentioning that, compared with the detected contents of AFM1 in the raw milk samples, the detected contents in dairy products are lower by one or two magnitude orders. This can be explained by the peculiarities of dairy production in Armenia, particularly considering that during production, a mix of locally produced raw milk from different regions was used, along with other raw materials (e.g., milk powder mainly imported to the country from the outside). Therefore, it can be assumed that mixing contaminated and non-contaminated raw milk, or other raw materials, may have contributed to the reduction in AFM1 levels in the final dairy products.

### 3.3. Consumption Levels of Raw Milk

According to the database on the national food balances for 2021 [28], the per capita consumption of raw milk is 677.1 g per day. Notable, this national per capita consumption rate includes data on all dairy products converted into fresh (raw) milk. Moreover, the per capita consumption data on raw milk apply to the general population of Armenia, including toddlers, children, and adolescents.

### 3.4. Consumption Levels of Dairy Products

The daily consumption data on the four common types of dairy products (Table 5) were obtained via a 24 h recall survey with 1400 adult respondents in Armenia.

In contrast to the data from the national food balances database, the 24 h recall survey data (Table 5) indicate average daily consumption rates of each studied dairy product among the entire studied adult population (i.e., all consumers) as well as in males and females.

### 3.5. Daily Intake and Risk Characterization of AFM1 in Raw Milk

The daily intake of AFM1 in raw milk was estimated considering the LB, MB, UB, and average levels of AFM1 in raw milk samples, along with the per capita consumption rate of fresh (raw) milk in Armenia (Table 6). This per capita consumption rate includes data on locally consumed dairy products converted to fresh (raw) milk. On the other hand, these figures cannot consider the high-level (e.g., 90th percentile) consumers of certain commodities.

Since AFM1 is considered to be potentially carcinogenic [36], no tolerable daily intake is set. The possible health risk for consumers is assessed by the margin of exposure (MOE) approach; the MOE does not quantify the risk but indicates whether an exposure scenario poses a health concern [35]. For carcinogenic substances, an MOE lower than 10,000 indicates a possible health concern. The HBGV for AFM1 was set by the EFSA CONTAM panel, considering a carcinogenic potency of 0.1 compared to the parent compound AFB1, i.e., 4 µg/kg bw per day [7].

The estimated MOE values for raw milk are consistently above the threshold of 10,000, even with the conservative UB scenario, thus not raising a health concern.

A worst-case scenario considered the average of the detected contents of AFM1 in raw milk samples, as it might occur following a hotspot of feed contamination. In such a scenario, the MOE value was 2213, below the threshold (10,000), indicating a public health concern.

Considering the study outcomes on the daily intake and risk characterization of AFM1 in raw milk, it is crucial to note that exposure to AFM1 in raw milk, even at levels below the national maximum level (0.5 μg/kg) set by the technical regulations, may still pose a public health concern. This indicates that the current regulatory level in Armenia does not fully guarantee the safety of the product and, consequently, the protection of consumer health.

### 3.6. Daily Intake and Risk Characterization of AFM1 in Dairy Products

Combining the average daily consumption of each dairy product, as well as the LB, MB, UB, and average levels of AFM1 in the products, the dietary exposure and induced possible health risks of AFM1 were estimated for males, females, and all consumers of the investigated dairy products (Table 7).

Overall, the results on dietary exposure showed that the estimated daily intake (DI) values of AFM1 for female consumers were slightly higher compared to those estimated for males and all consumers (Table 7). Nevertheless, all estimated DI values in the case of the four dairy products were at least 1000-fold lower compared to the DI values for raw milk. This difference can be explained by the fact that the consumption rates of dairy products in adults are significantly lower compared to the per capita consumption data for raw milk; in fact, the latter represent the consumption of the whole range of milk-based commodities by all citizens, including the younger age groups. Moreover, the reported AFM1 levels in dairy products were also considerably lower compared to those in the investigated raw milk. The resulting MOE values (Table 7) were well above the threshold of 10,000 in all cases.

The worst-case analysis considered a habitual consumer of both curd cheese and sour milk with the average AFM1 levels detected in this study (0.0063 and 0.001 μg/kg, respectively). Also, in this worst-case scenario, the MOE (Table 7) was above the threshold (5.5 × 10^6^, 4.5 × 10^6,^ and 4.90 × 10^6^ for males, females, and all consumers, respectively).

EFSA reported that no comprehensive dietary exposure assessment for aflatoxins is available in the EU. Nevertheless, in its latest scientific opinion on the risk assessment of aflatoxins, the EFSA reported that chronic dietary exposure to AFM1 for the adult population across European countries was estimated at maximums of 1.4 × 10^−4^ and 2.0 × 10^−4^ μg/kg/day in the lower bound (LB) and upper bound (UB) cases, respectively [7]. The figures for adults are still above the threshold of 10,000, yet they are quite close to it, suggesting that AFM1 contamination should be monitored and checked through the dairy production chain, starting from feeds and the farm [18,19].

There were mycotoxin-dedicated total diet studies (TDSs) in a few EU countries that also included AFM1 [45,46]. The UB exposures estimated in these TDSs were primarily driven by the LOQ values due to the high percentage of left-censored data [7], as was also the case in the present study conducted in Armenia.

Notable, the results on AFM1 exposure due to the consumption of dairy products in this study are much lower than those reported by other researchers [41,43,44,45].

The risk assessment results in this study (Table 7) showed that all estimated MOE values for AFM1 via dairy product consumption among the studied adult population are significantly higher than the threshold of 10,000, even when performing conservative worst-case estimates. According to the EFSA Scientific Committee, the calculated MOE reveals a low concern from a public health perspective [7].

## 4. Conclusions

This pilot research study is unique in the Caucasus region due to its approach to data analysis and its focus on aflatoxin-related issues, which have recently gained significant attention worldwide. For the first time in Armenia, this study estimated the dietary exposure and potential health risks related to AFM1 in raw milk and dairy products consumed by the adult population of the country.

The outcomes of the dairy product investigation showed that exposure to AFM1 is unlikely to raise any health concerns for Armenia’s adult population. However, based on data from the national residue monitoring program, the detected contents of AFM1 in raw milk samples were found to be below the nationally applicable maximum level of 0.5 µg/kg, while exceeding the EU maximum level of 0.05 µg/kg. While the rate of positive samples was low (7%), the AFM1 levels measured were definitely higher than those detected in countries with dietary and environmental features comparable to Armenia. A realistic worst-case estimate using the detected AFM1 values showed an MOE of 2200, well below 10,000, which raises public health concerns.

Our study highlights an important point: the maximum allowable level (0.5 µg/kg) of AFM1 set by the technical regulations and applied at the national level does not completely ensure the safety of the product and the protection of consumers’ health. In fact, the results obtained from the risk assessment indicate that exposure to AFM1 in raw milk, even at levels below the national maximum allowable level (0.5 µg/kg), can still pose a public health concern.

It is important to note that, in this study, the raw milk consumption data taken from the national food balances include the consumption of all dairy products converted to fresh (raw) milk and apply to the general population of Armenia, including toddlers, children, and adolescents. Meanwhile, the individually-based food consumption data (i.e., 24 h recall survey data) on dairy products only apply to the adult Armenian population. Considering the outcomes of this study and the fact that children constitute a significant group of milk product consumers, future studies on AFM1 should include them as well. Additionally, it is important to broaden the study’s scope by including other sources of affordable dairy products in Armenia (and in several other countries as well), such as farmer’s markets and other unofficial venues. A notable issue is that products sold in the farmer’s markets across different regions of the country undergo veterinary–sanitary inspections but are not examined for aflatoxin M1 contamination. Unlike formal markets or stores, unauthorized venues other than farmer’s markets may not undergo any form of inspection. So, due to the lack of proper monitoring and enforcement of safety standards, potential health risks associated with product consumption cannot be underestimated. This is especially true when considering that people with low purchasing power and socioeconomic status likely make up most of the customers in these unofficial and poorly controlled markets.

To conclude, this study provides valuable insights into understanding aflatoxin-related issues in the Caucasus area and establishes scientific bases for comprehensive studies on AFM1.

## Figures and Tables

**Table 1:** The detected contents (µg/kg) of AFM1 in raw milk.

Product	AFM1 (µg/kg)	Number of Positive Samples
Raw Milk	0.334	3 out of 42 (7.14%)
	0.150	
	0.090	

**Table 2:** The concentration (µg/kg) of AFM1 in milk in some Middle East countries.

Country	Type	Number of Positive Samples (%)	Concentration (µg/kg)		Reference
			Mean	Maximum	
Iran	Raw	2547 out of 3796 (67.1%)	0.056	0.068	[40]
Lebanon	Raw	28 out of 42 (66.67%)	0.041	0.078	
Turkey	Raw	73 out of 420 (17.38%)	0.083	0.164	

**Table 3:** The detected contents (µg/kg) of AFM1 in dairy products.

Dairy Products	AFM1 (µg/kg)	Number of Positive Samples
Pasteurized milk	ND	0 out of 10
Cheese	ND	0 out of 10
Sour cream	0.001	3 out of 10 (30%)
	0.001	
	0.001	
Curd cheese	0.005	4 out of 10 (40%)
	0.009	
	0.009	
	0.002	

Note: ND—non-detected.

**Table 4:** The concentrations (µg/kg) of AFM1 in milk products in some Mediterranean countries.

Country	Type	Number of Positive Samples (%)	Concentration (µg/kg)		Reference
			Mean	Maximum	
Greece	Pasteurized	70 out of 82 (85.37%)	0.010	0.05	[42]
	Conventional	75 out of 154 (48.7%)	0.009	0.013	
Spain	whole UHT *	68 out of 72 (94.44%)	0.0097	0.014	[41]
Lebanon	Milk and milk-based beverage	nr **	0.15	0.18	[44]

Note: *—ultra-heat treatment; **—not reported.

**Table 5:** Daily consumption (g/day) of dairy products among the Armenian adult population.

Dairy Products	Average Daily Consumption (g/day)			% of Consumers
	Male	Female	All Consumers	
Pasteurized milk	27.3	26.9	27.1	53.2
Cheese	21.1	17.6	18.8	86.5
Sour cream	16.7	16.2	16.4	85.5
Curd cheese	6.4	6.9	6.7	49.0

**Table 6:** Daily intake (DI) and margin of exposure (MOE) of AFM1 in raw milk.

Product Name	AFM1 LB/MB/UB * (μg/kg)		DI (μg/kg/day)	MOE
Raw milk	LB	0.0137	1.29 × 10^−4^	30,926
	MB	0.0243	2.30 × 10^−4^	17,361
	UB	0.035	3.31 × 10^−4^	12,068
	Average **	0.191	1.81 × 10^−3^	2213

Note: * At the lower bound (LB), <LOQ results were replaced with zero, at the upper bound (UB), <LOQ results were replaced with a value equal to LOQ, and at the middle bound (MB), the results were replaced with a value equal to LOQ/2. ** The average was calculated from the detected contents of AFM1 in three samples of raw milk.

**Table 7:** Daily intake (DI) and margin of exposure (MOE) of AFM1 in dairy products.

Dairy Products	AFM1 LB/MB/UB * (μg/kg)		DI (μg/kg/day)			MOE		
			Male	Female	All Consumers	Male	Female	All Consumers
Pasteurized Milk	LB	0	0	0	0	-	-	-
	MB	0.0005	1.8 × 10^−7^	2.0 × 10^−7^	1.89 × 10^−7^	2.27 × 10^7^	1.97 × 10^7^	2.11 × 10^7^
	UB	0.001	3.5 × 10^−7^	4.1 × 10^−7^	3.79 × 10^−7^	1.14 × 10^7^	9.83 × 10^6^	1.06 × 10^7^
	Average	<LOQ	-	-	-	-	-	-
Cheese	LB	0	0	0	0	-	-	-
	MB	0.0005	1.4 × 10^−7^	1.3 × 10^−7^	1.31 × 10^−7^	2.94 × 10^7^	3.01 × 10^7^	3.04 × 10^7^
	UB	0.001	2.7 × 10^−7^	2.7 × 10^−7^	2.63 × 10^−7^	1.47 × 10^7^	1.50 × 10^7^	1.52 × 10^7^
	Average	<LOQ	-	-	-	*-*	*-*	*-*
Sour cream	LB	0.0003	6.5 × 10^−8^	7.4 × 10^−8^	6.88 × 10^−8^	6.19 × 10^7^	5.44 × 10^7^	5.82 × 10^7^
	MB	0.0007	1.4 × 10^−7^	1.6 × 10^−7^	1.49 × 10^−7^	2.86 × 10^7^	2.51 × 10^7^	2.68 × 10^7^
	UB	0.001	2.2 × 10^−7^	2.5 × 10^−7^	2.29 × 10^−7^	1.86 × 10^7^	1.63 × 10^7^	1.74 × 10^7^
	Average **	0.001	2.2 × 10^−7^	2.5 × 10^−7^	2.29 × 10^−7^	1.86 × 10^7^	1.63 × 10^7^	1.74 × 10^7^
Curd cheese	LB	0.0025	2.1 × 10^−7^	2.6 × 10^−7^	2.35 × 10^−7^	1.95 × 10^7^	1.54 × 10^7^	1.70 × 10^7^
	MB	0.0028	2.3 × 10^−7^	2.9 × 10^−7^	2.63 × 10^−7^	1.74 × 10^7^	1.38 × 10^7^	1.52 × 10^7^
	UB	0.0031	2.5 × 10^−7^	3.2 × 10^−7^	2.91 × 10^−7^	1.57 × 10^7^	1.25 × 10^7^	1.37 × 10^7^
	Average **	0.0063	5.1 × 10^−7^	6.5 × 10^−7^	5.87 × 10^−7^	7.80 × 10^6^	6.18 × 10^6^	6.81 × 10^6^
Worst-case analysis			7.3 × 10^−7^	8.9 × 10^−7^	8.16 × 10^−7^	5.5 × 10^6^	4.5 × 10^6^	4.90 × 10^6^

Note: * At the lower bound (LB), <LOQ results were replaced with zero, at the upper bound (UB), < LOQ results were replaced with a value equal to LOQ, and at the middle bound (MB), the results were replaced with a value equal to LOQ/2. ** The average was calculated from the detected contents of AFM1 in the product.

## Data Availability

The original contributions presented in the study are included in the article, further inquiries can be directed to the corresponding author.
